# Baseline results of the first malaria indicator survey in Iran at the health facility level

**DOI:** 10.1186/1475-2875-10-319

**Published:** 2011-10-27

**Authors:** Ahmad Raiesi, Fatemeh Nikpour, Alireza Ansari-Moghaddam, Mansoor Ranjbar, Fatemeh Rakhshani, Mahdi Mohammadi, Aliakbar Haghdost, Rahim Taghizadeh-Asl, Mohammad Sakeni, Reza Safari, Mehdi Saffari

**Affiliations:** 1 Centre for Disease Control & Prevention, Ministry of Health and Medical, Education, Tehran, Iran; 2 Health Promotion Research Centre, Zahedan University of Medical Sciences, Zahedan, Iran; 3 United Nation Development Program, Tehran, Iran; 4 Physiology Research Centre, Kerman University of Medical Sciences, Kerman, Iran; 5 Province Health Centre, Zahedan University of Medical Sciences, Zahedan, Iran; 6 Province Health Centre & Deputy of Research, Hormozgan University of Medical Sciences, Bandar Abbas, Iran; 7 Province Health Centre, Kerman University of Medical Sciences, Kerman, Iran

## Abstract

**Background:**

Malaria continues to be a global public health challenge, particularly in developing countries. Delivery of prompt and effective diagnosis and treatment of malaria cases, detection of malaria epidemics within one week of onset and control them in less than a month, regular disease monitoring and operational classification of malaria are among the major responsibilities of the national malaria programme. The study was conducted to determine these indicators at the different level of primary health care facilities in malaria-affected provinces of Iran

**Methods:**

In this survey, data was collected from 223 health facilities including health centres, malaria posts, health houses and hospitals as well as the profile of all 5, 836 recorded malaria cases in these facilities during the year preceding the survey. Descriptive statistics (i.e. frequencies, percentages) were used to summarize the results and Chi square test was used to analyse data.

**Results:**

All but one percent of uncomplicated cases took appropriate and correctly-dosed of anti-malarial drugs in accordance to the national treatment guideline. A larger proportion of patients [85.8%; 95% CI: 84.8 - 86.8] were also given complete treatment including anti-relapse course, in line with national guidelines. About one third [35.0%; 95% CI: 33.6 - 36.4] of uncomplicated malaria cases were treated more than 48 hours after first symptoms onset. Correspondingly, half of severe malaria cases took recommended anti-malarial drugs for severe or complicated disease more than 48 hours of onset of first symptoms. The latter cases had given regular anti-malarial drugs promptly.

The majority of malaria epidemics [97%; 95% CI: 90.6 - 100] in study areas were detected within one week of onset, but only half of epidemics were controlled within four weeks of detection. Just half of target districts had at least one health facility/emergency site with adequate supply and equipment stocks. Nevertheless, only one-third of them [33% (95% CI: 0.00 - 67.8)] had updated inventory of malaria foci on quarterly basis.

**Conclusion:**

To sum up, malaria case management still constitutes a public health problem in Iran. Additionally, data suggest scarcity in management and evaluation of malaria foci, detection and control of malaria epidemics as well as assignment of emergency sites across different regions of the country. Consequently, massive and substantial investments need to be made at the Ministry of Health to coordinate national malaria control programmes towards achieving determined goals and targets.

## Background

Malaria is a global public health challenge, with about one million deaths each year and a further 250 million new cases of malaria diagnosed annually [1-3]. Interestingly, malaria have been distributed disproportionately such that the poorest countries in sub-Saharan Africa bearing about 85% of the burden of malaria morbidity and mortality in the world [1-3]. However, more than half of the populations of the Eastern Mediterranean Region are at potential risk of contracting malaria [1-3]. The Islamic Republic of Iran is one of the countries located in the Eastern Mediterranean Region with low malaria endemicity, with some regions having a reported API ranged from 0.14 to 8.74 per 1, 000 [3-6]. The south-eastern areas of Iran, including Sistan & Baluchestan (S&B), Hormozgan and the tropical part of Kerman provinces accounting for around 95% of all malaria cases in the country [4-6].

The Ministry of Health and Medical Education in Iran has focused its effort on recommended guidelines by World Health Organization (WHO) [7-9]. These strategies consist of early diagnosis and prompt effective anti-malarial treatment; detection of malaria epidemics within one week of onset and have it controlled in less than a month; operational classification of malaria foci on quarterly basis. Additionally, it is advised to assign emergency sites and provide them with adequate supply (i.e. spray pumps, thermal fogger machine both portable and vehicle mounted, insecticides for indoor residual spraying, insecticide for space spraying, insecticide-treated nets, anti-malarial drugs, rapid diagnostic tests, lancet, plain slide, alcohol pad, cotton, slide box, stationery) for urgent situation, such as outbreaks.

However, to date no comprehensive national malaria survey has been conducted to identify the coverage of these strategies in Iran. Additionally, the existing evidence about malaria indicators in the country, which has come mainly from routine surveillance data, is not sufficient to measure progress towards achieving national and regional goals and targets properly. Therefore, the present study was conducted to determine malaria indicators at the levels of health-treatment units and district health facilities and to investigate the quality of treatment of malaria cases in 20 malaria-affected districts in the three above-mentioned regions.

## Methods

### Sample design

Details of sampling methods have been described elsewhere [10]. Briefly, a comprehensive baseline survey conducted in Iran to evaluate malaria indicators in two levels: households and health facilities. The south-eastern provinces of Iran including 60% of the total population of S & B, the whole population of Hormozgan and 30% of the total population of Kerman provinces are considered as malaria endemic regions in Iran. These malarious regions with a population of about three million people formed the study population distributed in S&B, Hormozgan and Kerman provinces with an approximate proportion of 40%, 40% and 20%, respectively.

In a multistage random sampling, 125 clusters with an average size of 40 households were selected randomly from the study population. Firstly, sample size was divided based on the proportion of population in the three target provinces. Furthermore, since each province is, in turn, administratively subdivided into several districts and each district includes several rural and urban areas, the sample size of each province was divided based on the proportion of population in rural and urban areas. Secondly, all the health centers/units in the target districts in the three mentioned provinces were listed based on geographical regions and separately for urban and rural and then the populations were calculated cumulatively. At this stage, clusters and head-clusters (the first selected household as opening point for survey) were determined using systematic random sampling method. After that, trained personnel referred to the first household in every cluster and moved on their right side to cover the entire forty households in every cluster. Household information has been reported in a previously published article on malaria indicators at household level [10].

The survey also collected information from all health facilities in which at least one household had been selected for this study. Consequently, the profile of 5, 836 recorded malaria cases within included health facilities during the year preceding the survey were reviewed and data were extracted to evaluate malaria diagnosis and treatment. Furthermore, all the target district health centres were censuses to estimate malaria indicators at district level or health centres. Having this approach, data were collected from 223 health facilities in both rural and urban area of malaria endemic regions in Iran distributed in S & B, Hormozgan and Kerman provinces.

### Survey questionnaires

Three questionnaires were used for this part of survey: the health-caring units, patients' information in the health care services, and malaria indicators in district health centres. After investigating similar studies in other parts of the world [11-15] and also reviewing available questionnaires on the website of WHO [16-18], the organizers of this survey developed the questionnaires for the local situation and their completion instructions. Then questionnaires were pre-tested in the field and their results were discussed in several meetings. Afterwards, the questionnaires and their completion instructions were revised carefully to fulfill purpose of study. Finally, a meeting was held with the participation of the national programme manager for malaria, malaria project manager for round seven Global Fund malaria project, main investigators, a group of knowledgeable malaria experts from all three provinces. In this meeting, the contents of questionnaires were reviewed item by item and modified, if necessary. Then all the questionnaires and their completion instructions were finalized for the survey. Data were collected by trained health professionals.

### Data management and analysis

Data for malaria cases, health-treatment services and district health centres were gathered by teams of well-qualified data collectors through direct referring to the selected health facilities and filling in questionnaires, and checklists. Each team of data collectors comprised one health professional, one laboratory technician and team supervisor who were experienced and familiar with the basic concepts, objectives and technical terms of the project as well as one driver. Once all the information was collected and entered to the software of SPSS (Version 15) by trained data operators, analysis was done and descriptive statistics (i.e. frequencies, percentages) were used to estimate malaria indicators. Point estimates and 95% confidence intervals were estimated for all indicators. Relevant parametric or non-parametric statistical test, such as Pearson's Chi square test was used to determine association with a probability of committing a type 1 error (a) was set at 0.05.

### Evaluation the quality of project implementation

The quality of data collection was supervised and monitored daily by main investigators, provincial and district focal points during implementation of the project to ensure the quality and quantity of data collected by data collectors. Additionally, the quality of data entering was also monitored in two stages: in the primary stage, data file and its manual was prepared by skilled experts in statistics (MM & AAM). Then, data was entered by a team of trained data entry operators under the direct supervision of specialists in statistics and epidemiology (MM, AAM & AH). In the final stage, as data was analysed the information about every variable was controlled once more and if necessary was corrected by referring to the questionnaires.

### Ethical clearance

This study was approved by the research ethics committee of Zahedan University of Medical Sciences, Iran.

## Results

### Characteristics of study units

Data were collected from all 223 health facilities located in the selected districts to estimate health facility-based indicators, including detection and control of epidemics, management and evaluation of malaria foci and service delivery. Additionally, data were extracted from the profile of 5, 836 recorded malaria cases in aforementioned health centres during the year preceding the survey.

### Malaria case management

**Figure 1:**
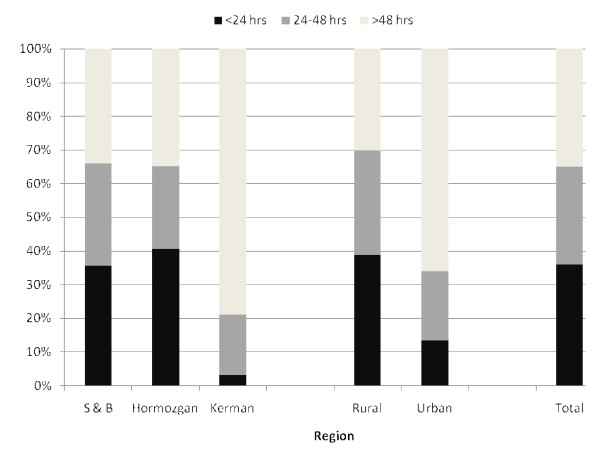
**Time interval between onset of symptoms and treatment of uncomplicated malaria cases**.

**Figure 2:**
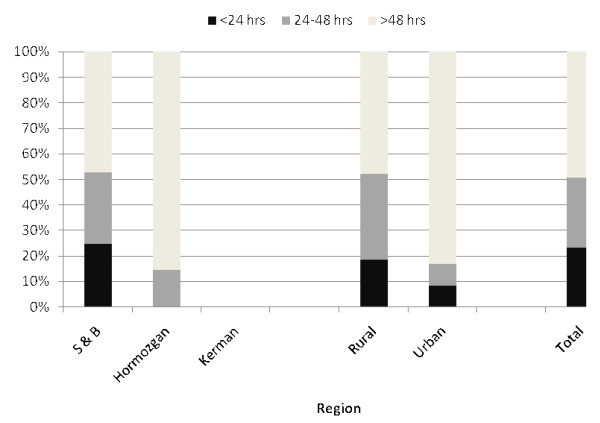
**Time interval between onset of symptoms and treatment of severe malaria cases**.

Generally, all but one percent of uncomplicated cases took appropriate and correctly-dosed of anti-malarial drugs in accordance to the national treatment guidelines. There is also evidence that a larger proportion of patients [85.8%; 95% CI: 84.8 - 86.8] were given complete treatment including anti-relapse course, in line with national guidelines whereas the remaining cases (14.2%) did not take anti-relapse course because either of immigration, or being less than five years of age or pregnancy that is primaquine is contraindicated. However, approximately two-third of uncomplicated (Figure 1) and half of severe (Figure 2) malaria cases were treated promptly on the same day or next day of 1st symptoms onset. It should be noted that all severe cases had given regular anti-malarial drugs promptly. Time interval between lab diagnosis and treatment were less than 24 hours for all reviewed malaria cases.

Notably, malaria patients both uncomplicated (Figure 1) and severe (Figure 2) cases in rural areas were more likely to receive medical attention and take an anti-malarial drug within 24 to 48 hours of disease onset (P for heterogeneity = 0.001). Apparently, two-third of uncomplicated and 83.3% (95% CI: 62.3% - 100%) of severe cases in urban areas of target districts sought medical attention and anti-malarial drug after 48 hours of 1^st ^symptoms onset. Subgroup analyses also suggest discrepancy in time interval between onset of symptoms and laboratory diagnosis/treatment of malaria patients across three surveyed provinces. Prompt treatment with anti-malarial drugs was highest for both uncomplicated and severe malaria cases in S&B region. On the contrary, treatment with anti-malarial drugs for uncomplicated patients in Kerman and severe in Hormozgan tended to be delayed.

### Malaria relapse history and mortality

There was no evidence of death due to malaria in the year preceding the survey. However, according to these findings, less than 10% of patients had a history of malaria relapse due to vivax malaria.

### Detection and control of epidemics

**Figure 3:**
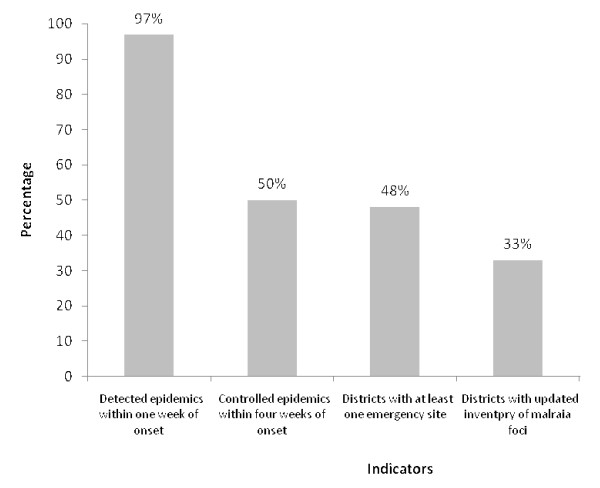
**Malaria indicators at health facilities**.

During the year preceding the survey, 42 malaria epidemics occurred in the country. Majority of these outbreaks happened in S& B province. Lack of detailed data regarding 11 epidemics precludes estimation of indicators on all recorded epidemics. Therefore, analysis was restricted on remaining 31 epidemics with adequate data. 97% of the epidemics (95% CI: 90.6 - 100)] in target districts detected within one week of onset by health staff. However, only half of them were controlled within four weeks of detection (Figure 3).

### Emergency sites with adequate supply and equipment

Generally speaking, there was no sufficient data to have a good judgment about the aforementioned indicators. However, 10 out of 21 sampled health centres [48%: (95% CI; 17.0 - 79.0)] had at least one assigned small emergency site with adequate supply and equipment stock to be used in outbreaks (Figure 3). Just in one district (Ghaleh-Ganj in Kerman); two emergency sites were identified to deal with outbreaks. Additionally, data suggest inconsistency in assignment of emergency sites across three provinces. Number of districts with at least one identified emergency sites were drastically higher in Kerman (100%) compared to the Hormozgan [44.4% (95% CI: 12.0 - 76.9) or S&B (0.0%) province.

### Management and evaluation of malaria foci

Only one-third of target districts [33% (95% CI: 0.00 - 67.8)] had updated inventory of malaria foci on quarterly basis (Figure 3). Furthermore, data shows that health centres in S&B or Kerman region [50% (95% CI: 10.0 - 90.0)] were more likely to make an inventory of malaria foci than those in Hormozgan [11.1 (95% CI: 0.00 - 100.0)].

## Discussion

This first comprehensive assessment of the coverage of the key malaria indicators at the level of health facilities in Iran highlights several important issues that need to be addressed urgently by Ministry of Health and Medical Education to achieve malaria control targets set by RBM partnership and Global Malaria Programme (GMP).

One of the specific measures to be applied in order to achieve malaria elimination is reducing the parasite reservoir through early diagnosis and treatment and use of efficacious medicines. Based on GMP targets [7-9], 80% of individuals suffering from uncomplicated or severe malaria should have access to and be able to use correct, affordable, and appropriate treatment within 24 hours. The present study demonstrated that the majority of both severe and uncomplicated malaria cases were given appropriate and complete treatment in line with national guideline. However, treatment was sought within 24 to 48 hours for two-third of uncomplicated and half of severe malaria cases which is far from the 80% target set by the RBM Partnership for 2010. Available documents from other parts of the world [11-15,19-21], with an exception for Morogoro Region (82.7%), have also shown that the proportion of patients receiving an appropriate anti-malarial timely and correctly-dosed were low and too far from the RBM targets for 2010.

Furthermore, data suggests striking differences in time interval between onset of symptoms and lab diagnosis/treatment of malaria patients across three surveyed provinces, which is consistent with the previous studies [11-15,19-21]. Interestingly, individuals in urban areas were more likely than those in rural areas to receive anti-malarial drugs or receive treatment promptly amongst the prior studies [11-15,19-21]. In contrast, malaria patients both uncomplicated and severe cases in rural areas were more likely to receive medical attention and take an anti-malarial drug within 24 to 48 hours of disease onset in the current study. The reason for this might be the attendance of health house in rural areas with local staff and adequate supply and equipment, which facilitate prompt diagnosis and treatment.

Prior studies [19] have also suggested that several factors related to the community and health system may hinder access to prompt and effective treatment. The community factors could be classified as cultural factors, the people's poor perceptions of quality of care, their lack of trust in health providers, and their perception of the effectiveness of drugs provided in government health facilities. Health system factors comprised of drug availability, provider practices such as careless of health workers in prescribing appropriate anti-malarial or doses to patients.

On the other hand, an interventional study in Tanzania [22] illustrated that improving understanding and treatment of malaria could lead to tangible improvements in terms of people's perception and actions for the treatment of malaria. Therefore, the Ministry of Health in Iran should invest adequately in above-mentioned mechanisms that may promote access to effective treatment.

Furthermore, the operational classification of malaria foci is one of the key components of GMP at the stage of malaria elimination. The transition of the functional status of malaria focus depending on the local situation and it might change constantly. According to the WHO classification [8], four categories of malaria foci are recognized including residual non-active; residual active; new potential and new active. Therefore, the status of every malaria focus must be reviewed on the quarterly basis and re-categorized when necessary. Consequently, the national malaria programme of Iran has also adopted this strategy during the past several years. However, the present study revealed that only one-third of target districts in Iran had updated inventory of malaria foci in accordance to the GMP.

Moreover, the centre for disease control and prevention in Iran aims to detect malaria epidemics (a majority of them are due to vivax malaria) within one week of onset and control them in less than a month, as advised by RBM programme [7-9]. Approximately, all the malaria epidemics with the available data in target districts detected within one week of onset by health staff, which is in line with GMP (80%). However, only half of them were controlled within four weeks of detection, which is lower than RBM/GMP goal. A recent review of epidemics in Africa [23] has also revealed delays in both epidemic detection and response up to 20 weeks, basically, due to poor case reporting and analysis or low use of public facilities. According to this study, epidemic controls were deficient and epidemic lasted between 15 - 36 weeks.

In comparison, Coleman *et al *[24] examined a three-tier malaria outbreak identification system using historical binomial thresholds to prompt early outbreak detection and response in South Africa. With this system, 100% health facilities reported outbreaks within 72 hours and appropriate response to all malaria outbreaks were achieved within 24 hours. Importantly, the binomial exact thresholds produced only one false weekly outbreak compared to 9 to 12 false weekly outbreaks by other recommended statistical approaches (i.e. mean + 2 SD) in the same study. These contradictions revealed the fact that malaria epidemics are multifactorial and difficult to predict correctly and timely only with the current advised methods. Therefore, research is needed to examine different methods for early identification of malaria outbreaks based on all relevant data, which in turn could facilitate timely response to epidemics.

Furthermore, health centres have proposed to assign and equip some of their health facilities with adequate supply (i.e. spray pumps, thermal fogger machine both portable and vehicle mounted, insecticides for indoor residual spraying, insecticide for space spraying, insecticide-treated nets, anti-malarial drugs, rapid diagnostic tests, lancet, plain slide, alcohol pad, cotton, slide box, stationery) for emergency situation such as outbreaks. The current findings showed that less than half of sampled health centres had at least one assigned small emergency site with adequate supply and equipment stock to be used in outbreak. Importantly, there was inconsistency in assignment of emergency sites across three provinces. Therefore, the present study indicates shortage and inconsistency in management and evaluation of malaria foci, detection and control of malaria epidemics as well as assignment of emergency sites across different regions of Iran.

## Conclusion

In conclusion, massive and substantial investments are needed to coordinate national malaria control programmes in Iran towards achieving determined goals and targets set by WHO. The first requirement is improving the quality of malaria case management. Secondly, health policy makers have to strength the epidemic preparedness and response capacity at the national level. More importantly, Ministry of Health should be ensured about the availability of nationally recommended anti-malarial drugs as well as other equipments stocks in all health facilities.

The national malaria control programme of Iran should also put an emphasis on robustness and activeness of surveillance systems coupled with vigorous community recruitment as the driving force to ensure increased access to treatment. Finally, future research should focus on examination of health worker practices regarding case management as well as epidemic identification and response.

## Competing interests

The authors declare that they have no competing interests.

## Authors' contributions

The overall implementation of the first malaria indicator survey in Iran including survey design, drafting the questionnaires, data management and analysis, report writing and manuscript preparation were the results of joint efforts by multiple individuals who are listed as co-authors of this paper. AR, AAM, FR, MM, MR, AH, FN and RTA made a substantial contribution on conception, design of study, coordination and supervision of data collection. MS, RS and MS gave a major contribution on preparing study questionnaires and data collection. MM and AAM analysed and interpreted data. AR, AAM and FN drafted the first version of manuscript. All authors have made extensive contribution into the review and finalization of this manuscript; they have all read and approved the final manuscript.

## References

[B1] World Health Organization 10 facts on malaria http://www.who.int/features/factfiles/malaria/en/index.html Accessed 18/10/2010

[B2] World Health Organization World malaria report 2009 Geneva, Switzerland

[B3] Sadrizadeh B Malaria in the World, in the Eastern Mediterranean region and in Iran Review article 2001. WHO/EMRO Report 1 13

[B4] Haghdoost AA Alexander N Cox J Modelling of malaria temporal variations in Iran Trop Med Int Health 2008 13 1501 1508 10.1111/j.1365-3156.2008.02166.x 19000157

[B5] Edrissian GH Malaria in Iran: Past and present situation Iranian J Parasitol 2006 1 1 14

[B6] Khalili MB Anvari-Tafti MH Sadeh M Epidemiological pattern of malarial disease in the province of Yazd, Iran (Since 1986-2006) World Journal of Medical Sciences 2009 4 41 45

[B7] Roll Back Malaria Partnership Roll Back Malaria Global Strategic plan 2005 - 2015 http://www.rollbackmalaria.org/forumV/globalstrategicplan.htm Accessed: 9/3/2010

[B8] World Health Organization Malaria elimination: a field manual for low and moderate endemic countries Geneva, Switzerland

[B9] Roll Back Malaria Partnership The Global Malaria Action Plan: for a malaria-free world 2008 Geneva, Switzerland

[B10] Mohammadi M Ansari-Moghaddam A Raiesi A Rakhshani F Nikpour F Haghdost A Ranjbar M Taghizadeh-Asl R Sakeni M Safari R Saffari M Baseline results of the first malaria indicator survey in Iran at household level Malar J 2011 10 277 10.1186/1475-2875-10-277 21939505 PMC3184285

[B11] Noor AM Djibouti National Malaria Indicator Survey 2008-2009 2009 Submitted to the World Health Organization, Eastern Mediterranean Regional Office, Cairo, Egypt

[B12] Ministry of Health (MOH) [Rwanda], National Institute of Statistics of Rwanda (NISR), and ICF Macro Rwanda Interim Demographic and Health Survey 2007-08 2009 Calverton, Maryland, USA: MOH, NISR, and ICF Macro

[B13] Consultoria de Serviços e Pesquisas-COSEP Lda., Consultoria de Gestão e Administração em Saúde-Consaúde Lda. [Angola], and Macro International Inc Angola Malaria Indicator Survey 2006-07 2007 Calverton, Maryland: COSEP Lda., Consaúde Lda., and Macro International Inc

[B14] National Malaria Control Program (NMCP) [Liberia], Ministry of Health and Social Welfare, Liberia Institute of Statistics and Geo-Information Services (LISGIS), and ICF Macro Liberia Malaria Indicator Survey 2009 2009 Monrovia, Liberia: NMCP, LISGIS, and ICF Macro

[B15] National Malaria Control Center [Zambia], Ministry of Health; Central Statistical Office; Malaria Control and Evaluation Partnership in Africa (MACEPA), a program at PATH; the United States President's Malaria Initiative; the World Bank; UNICEF; the World Health Organization; and the University of Zambia Zambia National Malaria Indicator Survey 2008 Lusaka, Zambia

[B16] Roll Back Malaria Monitoring and Evaluation Reference Group, World Health Organization, United Nations Children's Fund, MEASURE DHS, MEASURE Evaluation, and U.S. Centers for Disease Control and Prevention Malaria Indicator Survey: Basic documentation for survey design and implementation 2005 Calverton, Maryland: MEASURE Evaluation

[B17] Monitoring and Evaluation Toolkit HIV/AIDS, Tuberculosis, and Malaria The Global Fund to Fight AIDS, Tuberculosis & Malaria Geneva, Switzerland http://www.theglobalfund.org ISBN 92-9224-029-3.2006, Accessed:9/3/201022036298

[B18] Roll Back Malaria, MEASURE Evaluation, World Health Organization, UNICEF Guidelines for Core Population Coverage Indicators for Roll Back Malaria: To Be Obtained from Household Surveys 2006 MEASURE Evaluation: Calverton, Maryland

[B19] Chuma J Abuya T Memusi D Juma E Akhwale W Ntwiga J Nyandigisi A Tetteh G Shretta R Amin A Reviewing the literature on access to prompt and effective malaria treatment in Kenya: implications for meeting the Abuja targets Malar J 2009 8 243 10.1186/1475-2875-8-243 19863788 PMC2773788

[B20] Hetzel MW Obrist B Lengeler C Msechu JJ Nathan R Dillip A Makemba AM Mshana C Schulze A Mshinda H Obstacles to prompt and effective malaria treatment lead to low community-coverage in two rural districts of Tanzania BMC Public Health 2008 8 317 10.1186/1471-2458-8-317 18793448 PMC2564938

[B21] Jima D Getachew A Bilak H Steketee RW Emerson PM Graves PM Gebre T Reithinger R Hwang J Ethiopia Malaria Indicator Survey Working Group Malaria indicator survey 2007, Ethiopia: coverage and use of major malaria prevention and control interventions Malar J 2010 9 58 10.1186/1475-2875-9-S2-P58 20178654 PMC2841196

[B22] Alba S Dillip A Hetzel MW Mayumana L Mshana C Makemba A Alexander M Obrist B Schulze A Kessy F Mshinda H Lengeler C Improvements in access to malaria treatment in Tanzania following community, retail sector and health facility interventions -- a user perspective Malar J 2010 9 163 10.1186/1475-2875-9-163 20550653 PMC2910017

[B23] Checchi F Cox J Balkan S Tamrat A Priotto G Alberti KP Zurovac D Guthmann JP Malaria epidemics and interventions, Kenya, Burundi, Southern Sudan, and Ethiopia, 1999-2004 Emerg Infect Dis 2006 12 1477 1485 17176560 10.3201/eid1210.060540 PMC3290957

[B24] Coleman M Coleman M Abuza AM Gerdalize K Coetzee M Durrheim DN Evaluation of an operational malaria outbreak identification and response system in Mpumalanga Province, South Africa Malar J 2008 7 69 10.1186/1475-2875-7-69 18439307 PMC2405804

